# Supplementary material for: Where the wild bees are: Birds improve indicators of bee richness
Source: PLoS One. 2025 Apr 23;20(4):e0321496. doi: 10.1371/journal.pone.0321496 (PMC12017907; doi:10.1371/journal.pone.0321496)
Supplement: S1 File — (PDF) [file pone.0321496.s001.pdf]

SUPPLEMENTAL MATERIAL

S1. Standardizing bird abundance using residuals

Bird abundance within each grid cell was standardized separately for each species and dataset. In the first stage of this process, we wanted to account for variation in effort across checklists. We first modeled bird count as a function of survey duration within a Generalized Additive Model. This function describes how bird counts on checklists varied with duration. For most species, the maximum count is reached for checklists of intermediate duration, beyond which counts tend to decline. Although this seems counterintuitive, it is a consistent pattern in many participatory science datasets that are likely to reflect different behavior of observers or different types of environments associated with longer duration checklists. We wanted to estimate this maximum in order to determine the survey duration (up to 300 minutes maximum) associated with the greatest counts for each species. Survey durations with the maximum counts ranged from 142 to 300 minutes across all the bird species using the semi-structured dataset and 146 to 300 minutes using the structured dataset.

In the second step, we used a log-link poisson Generalized Additive Model, to model bird counts per checklist as a function of effort variables that are likely to affect the observed counts: survey duration, survey distance, the time of the day when the survey started, and the protocol (stationary or traveling). The response variable in the models was the count of the bird species on each checklist, with the predictors being discretized smooth functions of the effort variables. We then calculated the expected count of the bird on each checklist (i.e. the fitted value from a prediction from the GAM model for each set of predictor variables). Next, we took the residual for each checklist: which would be high if the checklist reported more birds of the species than expected once accounting for the effort, and negative if the checklist reported fewer birds of the species than expected once accounting for the checklist effort. We then took the mean residual for all checklists within each grid cell. Positive values indicated when grid cells had higher numbers of that bird species than the average based on effort, whereas negative numbers indicated lower than average counts. This average residual per grid cell was used as a measure of bird abundance per species that was used in subsequent models of bee richness.
