# Supplementary material for: Where the wild bees are: Birds improve indicators of bee richness
Source: PLoS One. 2025 Apr 23;20(4):e0321496. doi: 10.1371/journal.pone.0321496 (PMC12017907; doi:10.1371/journal.pone.0321496)
Supplement: S2 File — (PDF) [file pone.0321496.s002.pdf]

SUPPLEMENTAL MATERIAL

S2. List of bird species excluded from analysis

The following species were excluded from the analysis as they are considered flyover species and could not be linked to the local habitat: American Kestrel, Bald Eagle, Broad-winged Hawk, Chimney Swift, Common Nighthawk, Cooper's Hawk, Red-shouldered Hawk, Red-tailed Hawk, Turkey Vulture, Black Vulture.
