# Supplementary material for: Where the wild bees are: Birds improve indicators of bee richness
Source: PLoS One. 2025 Apr 23;20(4):e0321496. doi: 10.1371/journal.pone.0321496 (PMC12017907; doi:10.1371/journal.pone.0321496)
Supplement: S3 Table — List of bird species and their scientific names, considered in the analysis. Only bird species with a prevalence (Prev) of 20% or greater and a breeding distribution covering at least 40% of the respective study region were included: the semi-structured dataset encompassed the eastern half of the U.S., while the structured dataset focused on several eastern states. Bird species selected in at least one of the 100 models for both dataset analyses are listed. (PDF) [file pone.0321496.s003.pdf]

SUPPLEMENTAL MATERIAL

S3. Bird species used in semi-structured and structured dataset analysis

Table S3. List of bird species and their scientific names, considered in the analysis. Only bird species with a prevalence (Prev) of 20% or greater and a breeding distribution covering at least 40% of the respective study region were included: the semi-structured dataset encompassed the eastern half of the U.S., while the structured dataset focused on several eastern states. Bird species selected in at least one of the 100 models for both dataset analyses are listed.

		Semi-structured data models			Structured data models		
**English Name**	**Scientific Name**	**Prev**	**Birds & land cover types**	**Birds only**	**Prev**	**Birds & land cover types**	**Birds only**
Acadian Flycatcher	*Empidonax virescens*	x	.	.	x	.	.
American Crow	*Corvus brachyrhynchos*	x	x	x	x	.	x
American Goldfinch	*Spinus tristis*	x	.	x	x	.	.
American Redstart	*Setophaga ruticilla*	x	x	x	x	.	.
American Robin	*Turdus migratorius*	x	.	x	x	.	.
Baltimore Oriole	*Icterus galbula*	x	.	.	x	.	.
Barn Swallow	*Hirundo rustica*	x	.	.	x	x	x
Barred Owl	*Strix varia*	x	.	.	.	.	.
Belted Kingfisher	*Megaceryle alcyon*	x	.	.	x	.	x
Black-and-white Warbler	*Mniotilta varia*	x	.	.	x	.	.
Black-billed Cuckoo	*Coccyzus erythropthalmus*	x	.	.	.	.	.
Black-capped Chickadee	*Poecile atricapillus*	x	x	x	.	.	.
Blue-gray Gnatcatcher	*Polioptila caerulea*	x	.	.	x	.	.
Blue Grosbeak	*Passerina caerulea*	x	.	x	x	.	.
Blue Jay	*Cyanocitta cristata*	x	x	x	x	x	x
Bobolink	*Dolichonyx oryzivorus*	x	.	.	.	.	.
Brown-headed Cowbird	*Molothrus ater*	x	.	.	x	x	x
Brown Thrasher	*Toxostoma rufum*	x	.	x	x	x	x
Canada Goose	*Branta canadensis*	x	.	.	x	.	x
Carolina Chickadee	*Poecile carolinensis*	x	.	.	x	.	.
Carolina Wren	*Thryothorus ludovicianus*	x	x	x	x	.	.
Cedar Waxwing	*Bombycilla cedrorum*	x	.	.	x	.	.
Chipping Sparrow	*Spizella passerina*	x	x	x	x	x	x
Cliff Swallow	*Petrochelidon pyrrhonota*	x	.	x	.	.	.
Common Grackle	*Quiscalus quiscula*	x	.	.	x	.	.
Common Yellowthroat	*Geothlypis trichas*	x	x	x	x	x	x
Dickcissel	*Spiza americana*	x	x	x	.	.	.

Downy Woodpecker	*Dryobates pubescens*	x	x	x	x	.	.
Eastern Bluebird	*Sialia sialis*	x	.	.	x	x	x
Eastern Kingbird	*Tyrannus tyrannus*	x	.	.	x	.	.
Eastern Meadowlark	*Sturnella magna*	x	.	.	.	.	.
Eastern Phoebe	*Sayornis phoebe*	x	.	.	x	.	.
Eastern Towhee	*Pipilo erythrophthalmus*	x	.	.	x	.	x
Eastern Wood-Pewee	*Contopus virens*	x	.	.	x	x	x
European Starling	*Sturnus vulgaris*	x	x	.	x	.	.
Field Sparrow	*Spizella pusilla*	x	.	.	x	.	.
Fish Crow	*Corvus ossifragus*	.	.	.	x	x	x
Grasshopper Sparrow	*Ammodramus savannarum*	x	.	.	x	.	.
Gray Catbird	*Dumetella carolinensis*	x	x	x	x	x	x
Great Blue Heron	*Ardea herodias*	x	.	.	x	x	x
Great Crested Flycatcher	*Myiarchus crinitus*	x	.	.	x	.	.
Green Heron	*Butorides virescens*	x	.	.	x	x	x
Hairy Woodpecker	*Dryobates villosus*	x	.	.	x	.	x
House Finch	*Haemorhous mexicanus*	.	.	.	x	x	x
House Sparrow	*Passer domesticus*	x	.	x	.	.	.
House Wren	*Troglodytes aedon*	x	.	.	x	x	x
Indigo Bunting	*Passerina cyanea*	x	.	.	x	x	x
Killdeer	*Charadrius vociferus*	x	.	.	x	.	.
Louisiana Waterthrush	*Parkesia motacilla*	x	.	.	.	.	.
Mallard	*Anas platyrhynchos*	x	.	.	x	.	x
Mourning Dove	*Zenaida macroura*	x	.	.	x	.	.
Northern Cardinal	*Cardinalis cardinalis*	x	x	x	x	.	x
Northern Flicker	*Colaptes auratus*	x	.	.	x	.	.
Northern Mockingbird	*Mimus polyglottos*	x	.	.	x	.	.
Northern Parula	*Setophaga americana*	x	.	.	x	.	.
Northern Rough-winged Swallow	*Stelgidopteryx serripennis*	x	.	.	x	x	x
Orchard Oriole	*Icterus spurius*	x	x	x	x	x	.
Ovenbird	*Seiurus aurocapilla*	x	.	.	x	x	x
Pileated Woodpecker	*Dryocopus pileatus*	x	.	.	x	.	x
Pine Warbler	*Setophaga pinus*	x	.	.	x	x	.
Prairie Warbler	*Setophaga discolor*	.	.	.	x	.	.
Purple Martin	*Progne subis*	x	.	.	x	x	x
Red-bellied Woodpecker	*Melanerpes carolinus*	x	x	x	x	x	.
Red-eyed Vireo	*Vireo olivaceus*	x	.	.	x	.	.
Red-headed Woodpecker	*Melanerpes erythrocephalus*	x	.	.	.	.	.
Red-winged Blackbird	*Agelaius phoeniceus*	x	.	.	x	x	x
Rose-breasted Grosbeak	*Pheucticus ludovicianus*	x	x	x	.	.	.
Ruby-throated Hummingbird	*Archilochus colubris*	x	x	x	x	.	.

Scarlet Tanager	*Piranga olivacea*	x	x	x	x	.	.
Song Sparrow	*Melospiza melodia*	x	.	x	x	.	x
Summer Tanager	*Piranga rubra*	.	.	.	x	x	.
Tree Swallow	*Tachycineta bicolor*	x	.	.	x	.	x
Tufted Titmouse	*Baeolophus bicolor*	x	.	.	x	x	.
Warbling Vireo	*Vireo gilvus*	x	x	x	x	.	.
White-breasted Nuthatch	*Sitta carolinensis*	x	x	x	x	.	x
White-eyed Vireo	*Vireo griseus*	x	.	.	x	.	.
Wild Turkey	*Meleagris gallopavo*	x	.	.	x	.	.
Willow Flycatcher	*Empidonax traillii*	x	.	.	.	.	.
Wood Duck	*Aix sponsa*	x	.	.	x	.	x
Wood Thrush	*Hylocichla mustelina*	x	.	x	x	x	x
Yellow-billed Cuckoo	*Coccyzus americanus*	x	.	x	x	.	.
Yellow-breasted Chat	*Icteria virens*	.	.	.	x	.	.
Yellow-throated Vireo	*Vireo flavifrons*	x	x	.	x	x	x
Yellow Warbler	*Setophaga petechia*	x	.	.	x	.	.
