# Supplementary material for: Where the wild bees are: Birds improve indicators of bee richness
Source: PLoS One. 2025 Apr 23;20(4):e0321496. doi: 10.1371/journal.pone.0321496 (PMC12017907; doi:10.1371/journal.pone.0321496)
Supplement: S4 Table — List of land cover types considered in the analysis. Only land cover types covering 20% or more of their respective study region were included (Prev): the semi-structured dataset encompassed the eastern half of the U.S., while the structured dataset focused on several eastern states. Land cover types selected in at least one of the 100 models for both dataset analyses are listed. (PDF) [file pone.0321496.s004.pdf]

SUPPLEMENTAL MATERIAL

S4. Land cover types used in semi-structured and structured dataset analysis

Table S4. List of land cover types considered in the analysis. Only land cover types covering 20% or more of their respective study region were included (Prev): the semi-structured dataset encompassed the eastern half of the U.S., while the structured dataset focused on several eastern states. Land cover types selected in at least one of the 100 models for both dataset analyses are listed.

	Semi-structured data models			Structured data models		
**Land cover types**	**Prev**	**Birds & land cover types**	**Land cover types only**	**Prev**	**Birds & land cover types**	**Land cover types only**
Alfalfa	x	x	x	x	x	x
Asparagus	.	.	.	.	.	.
Barren	x	x	x	x	.	x
Bean	x	.	x	x	.	x
Berries	.	.	.	.	.	.
Buckwheat	.	.	.	.	.	.
Christmas trees	.	.	.	.	.	.
Citrus	.	.	.	.	.	.
Coniferous forest	x	.	x	x	x	x
Corn	x	x	x	x	.	x
Cotton	.	.	.	.	.	.
Cucurbits	.	.	.	.	.	.
Deciduous forest	x	x	x	x	.	x
Developed open space	x	.	x	x	.	x
Double crop	x	x	x	x	.	x
Flowers	.	.	.	.	.	.
Grain	x	.	x	x	x	x
Grapes	.	.	.	.	.	.
Grass	.	.	x	.	.	x
Grass pasture	x	x	x	x	.	x
Herbaceous wetland	x	.	x	x	x	x
Herbs	.	.	.	.	.	.
Idle cropland	x	x	x	x	.	x
Melons	.	.	.	.	.	.
Mixed forest	x	.	x	x	x	x
Nuts	.	.	.	.	.	.
Oilseed	.	.	.	.	.	.
Olives	.	.	.	.	.	.

Open water	x	x	x	x	.	x
Orchard	.	.	x	.	.	.
Other crops	.	.	.	.	.	.
Root vegetables	.	.	.	.	.	.
Shrubland	x	.	x	x	.	x
Solanums	.	.	.	.	.	.
Strawberries	.	.	.	.	.	.
Tobacco	.	.	.	.	.	.
Tree crops	.	.	.	.	.	.
Urban high-density	x	.	x	x	.	x
Urban low-density	x	x	x	x	.	x
Urban medium-density	x	.	x	x	.	x
Vegetables	.	.	.	.	.	.
Vegetables and fruits	.	.	.	.	.	.
Watermelons	.	.	.	.	.	.
Wildflowers	.	.	.	.	.	.
Woody wetland	x	.	x	x	.	x
