# Supplementary material for: Where the wild bees are: Birds improve indicators of bee richness
Source: PLoS One. 2025 Apr 23;20(4):e0321496. doi: 10.1371/journal.pone.0321496 (PMC12017907; doi:10.1371/journal.pone.0321496)
Supplement: S5 File — Description of the process used. Includes comparison of the number of predictors used, resulting model fit and validation r-squared, predictors selected, and associated estimates per methodology tested. (PDF) [file pone.0321496.s005.pdf]

SUPPLEMENTAL MATERIAL

S5. Two-step process to select relevant predictors of bee richness

Because computational time increased non-linearly with the number of variables, it was impractical to use the Bayesian model selection procedure with a high number of predictor variables. To overcome this constraint, we employed a two-step process to first select the predictors used in the models to those most associated with bee richness. In order to ensure that this two-step process did not influence the results, for a subset of the models we directly compared the outcomes of this two-step process with those of running a model incorporating all predictors simultaneously. This assessment was conducted on a subset of 78 of 100 variables in the original dataset. This validation confirmed that the two-step process did not influence the results and so we used this for the main results in this paper.

In the initial step, we considered 78 bird species and land cover predictors. We divided the dataset into three subsets, each containing 26 predictors, and then selected the best three predictors in each subset, employing the same Bayesian variable selection methodology used in the model with all 78 predictors. We repeated this process by randomly shuffling the predictor list 1000 times, generating subsets, and selecting the best three predictors each time (we also experimented with using five predictors). We retained all predictors that were chosen at least once across all subsets. These retained predictors were subsequently utilized in the second step to identify the ten predictors most strongly correlated with bee richness. We calculated the model fit and performed a five-fold validation for this model. The results of this assessment indicate that the two-step process yielded the same predictors, predictor estimates, model fit, and validation R-squared as if we had used all predictors simultaneously. However, this two-step process significantly reduced the time required to extract these results, taking only 5 minutes as opposed to several days for the full model.

Table S5. Comparison of the number of predictors used, resulting model fit and validation r-squared, predictors selected, and associated estimates per methodology tested.

Model / process	Whole model	Whole model	Two-step process	Two-step process
**Scaling of predictors**	Not scaled	Scaled	Scaled	Scaled
**Total # of predictors considered**	78	78	78	78
**# grid cells**	571	571	571	571
**# predictors selected per subset**	.	.	5	3
**# predictors retained in two-step process**	.	.	51	41
**# best predictors selected**	10	10	10	10
**Model fit (r^2)**	0.265	0.265	0.265	0.265
**Five-fold validation (r^2)**	0.224	0.224	0.224	0.224
**Predictors**	**Predictor estimates and significance level**			
(Intercept)	0.2412	1.2353 ***	1.2353 ***	1.2353 ***
AmericanRobin	-0.1488 *	-0.1956 *	-0.1956 *	-0.1956 *
BrownThrasher	0.6347 ***	0.2482 ***	0.2482 ***	0.2482 ***
HairyWoodpecker	-0.5625 **	-0.1884 **	-0.1884 **	-0.1884 **
pctBean	0.0260 *	0.1680 *	0.1680 *	0.1680 *
pctDeciForest	0.0150 ***	0.2837 ***	0.2837 ***	0.2837 ***
pctHerbWetland	0.0160 *	0.1569 *	0.1569 *	0.1569 *
pctMixedForest	0.0687 ***	0.5343 ***	0.5343 ***	0.5343 ***
pctWoodyWetland	0.0338 ***	0.3773 ***	0.3773 ***	0.3773 ***
RubyThroatedHummingbird	-0.6614 **	-0.2251 **	-0.2251 **	-0.2251 **
WoodThrush	0.4635 **	0.2297 **	0.2297 **	0.2297 **
