# Supplementary material for: Where the wild bees are: Birds improve indicators of bee richness
Source: PLoS One. 2025 Apr 23;20(4):e0321496. doi: 10.1371/journal.pone.0321496 (PMC12017907; doi:10.1371/journal.pone.0321496)
Supplement: S6 Fig — Includes two plots: 1) Correlation between observed and predicted bee richness using the semi-structured dataset for 2,501 locations across the eastern half of the U.S., 2007–2021. The yellow contour lines represent the density of points and the red line the linear correlation, 2) Correlation between observed and predicted bee richness using the structured dataset for 194 locations across the eastern U.S., 2011–2015. The blue contour lines represent the density of points and the red line the linear correlation. (PDF) [file pone.0321496.s006.pdf]

SUPPLEMENTAL MATERIAL

S6. Plots of the observed and predicted bee richness for the large-scale and small-scale analysis using land cover types and bird data model

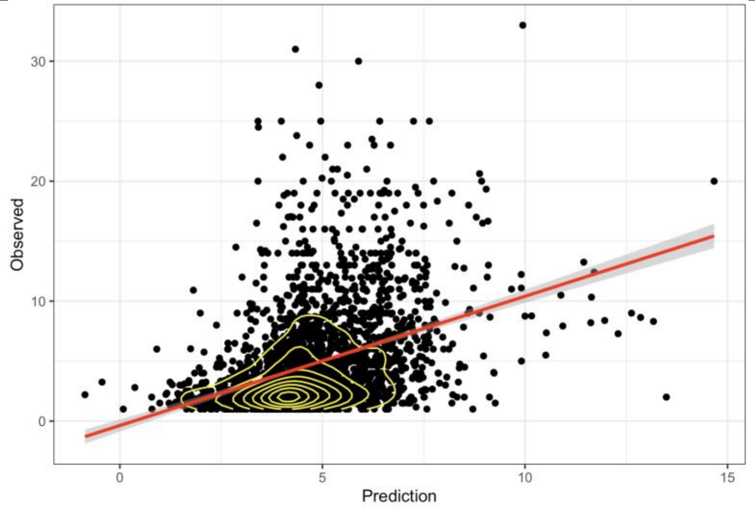

Fig. S6.1. Correlation between observed and predicted bee richness using the semi-structured dataset for 2,501 locations across the eastern half of the United-State, 2007 to 2021. The yellow contour lines represent the density of points and the red line the linear correlation.

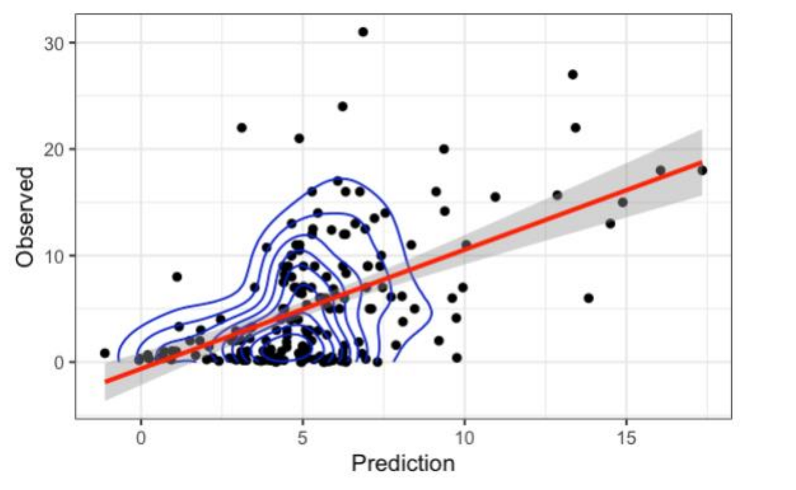

Fig. S6.2. Correlation between observed and predicted bee richness using the structured dataset for 194 locations across the eastern United-State, 2011 to 2015. The blue contour lines represent the density of points and the red line the linear correlation.
