# Supplementary material for: Where the wild bees are: Birds improve indicators of bee richness
Source: PLoS One. 2025 Apr 23;20(4):e0321496. doi: 10.1371/journal.pone.0321496 (PMC12017907; doi:10.1371/journal.pone.0321496)
Supplement: S7 Table — List of predictors selected at least once in each of the 100 models per variables set - land cover types only, birds only, and birds and land cover types - and their associated mean estimate, standard deviation in the estimates, and number of models in which they were selected. Predictor variables are sorted according to frequency of inclusion in “birds & land cover” models. (PDF) [file pone.0321496.s007.pdf]

SUPPLEMENTAL MATERIAL

S7. Structured dataset results

Table S7. List of predictors selected at least once in each of the 100 models per variables set - land covers only, birds only, and birds & land covers - and their associated mean estimate, standard deviation in the estimates, and number of models in which they were selected. Predictor variables are sorted according to frequency of inclusion in "birds & land cover" models.

	**Mean of estimates, SD (# of models)**		
**Predictor variables**	**Land cover only**	**Birds only**	**Birds & land cover**
Intercept	5.32, 0.00 (100)	5.32, 0.00 (100)	5.32, 0.00 (100)
Grain	1.59, 0.09 (100)	.	1.43, 0.09 (100)
Gray Catbird, *Dumetella carolinensis*	.	1.82, 0.20 (100)	1.34, 0.19 (100)
Yellow-throated Vireo, *Vireo flavifrons*	.	-0.96, 0.10 (99)	-0.98, 0.11 (97)
Green Heron, *Butorides virescens*	.	-0.92, 0.12 (72)	-0.92, 0.09 (93)
Brown Thrasher, *Toxostoma rufum*	.	-0.96, 0.11 (89)	-0.96, 0.14 (89)
Mixed forest	-0.95, 0.03 (100)	.	-0.81, 0.09 (84)
Chipping Sparrow, *Spizella passerina*	.	0.91, 0.13 (85)	0.85, 0.11 (76)
Brown-headed Cowbird, *Molothrus ater*	.	0.64, 0.09 (14)	0.76, 0.09 (58)
Fish Crow, *Corvus ossifragus*	.	-0.97, 0.13 (85)	-0.83, 0.14 (50)
Red-winged Blackbird, *Agelaius phoeniceus*	.	-1.24, 0.24 (65)	-1.02, 0.22 (43)
Purple Martin, *Progne subis*	.	-0.84, 0.14 (38)	-0.77, 0.11 (38)
Barn Swallow, *Hirundo rustica*	.	1.25, 0.21 (86)	1.09, 0.23 (35)
House Wren, *Troglodytes aedon*	.	-0.99, 0.17 (73)	-0.74, 0.08 (33)
Blue Jay, *Cyanocitta cristata*	.	-0.80, 0.09 (43)	-0.64, 0.07 (18)
Eastern Wood-Pewee, *Contopus virens*	.	-0.44 (1)	-0.73, 0.15 (16)

Great Blue Heron, *Ardea herodias*	.	-0.39 (1)	-0.63, 0.11 (15)
Coniferous forest	-0.15, 0.04 (10)	.	-0.70, 0.03 (13)
Eastern Bluebird, *Sialia sialis*	.	-0.66, 0.08 (13)	-0.65, 0.14 (11)
House Finch, *Haemorhous mexicanus*	.	-0.61, 0.12 (6)	-0.57, 0.09 (8)
Red-bellied Woodpecker, *Melanerpes carolinus*	.	.	-0.64, 0.04 (6)
Herbaceous wetland	-0.51, 0.04 (100)	.	-0.54, 0.08 (3)
Alfalfa	-0.33, 0.02 (65)	.	-0.64, 0.06 (3)
Northern Rough-winged Swallow, *Stelgidopteryx serripennis*	.	0.70, 0.11 (30)	0.48, 0.03 (2)
Summer Tanager, *Piranga rubra*	.	.	0.69, 0.33 (2)
Indigo Bunting, *Passerina cyanea*	.	0.62, 0.21 (7)	0.47 (1)
Common Yellowthroat, *Geothlypis trichas*	.	-0.69, 0.33 (2)	-0.57 (1)
Wood Thrush, *Hylocichla mustelina*	.	0.42 (1)	1.17 (1)
Ovenbird, *Seiurus aurocapilla*	.	-0.28 (1)	-1.31 (1)
Orchard Oriole, *Icterus spurius*	.	.	-0.49 (1)
Tufted Titmouse, *Baeolophus bicolor*	.	.	-0.51 (1)
Pine Warbler, *Setophaga pinus*	.	.	-0.79 (1)
Double crop	-0.48, 0.10 (98)	.	.
Urban medium-density	0.48, 0.10 (91)	.	.
Woody wetland	-0.42, 0.08 (91)	.	.
Shrubland	0.36, 0.04 (80)	.	.
Open water	-0.30, 0.03 (79)	.	.
Grass	-0.23, 0.02 (43)	.	.
Deciduous forest	0.27, 0.05 (32)	.	.

Corn	0.31, 0.06 (30)	.	.
Barren	-0.16, 0.02 (18)	.	.
Developed open space	-0.20, 0.03 (16)	.	.
Urban high-density	-0.13, 0.22 (14)	.	.
Urban low-density	-0.04, 0.25 (11)	.	.
Bean	-0.17, 0.09 (10)	.	.
Grass pasture	0.10, 0.09 (7)	.	.
Idle cropland	-0.03, 0.02 (5)	.	.
Mallard, *Anas platyrhynchos*	.	0.85, 0.13 (58)	.
Song Sparrow, *Melospiza melodia*	.	-0.75, 0.12 (14)	.
Hairy Woodpecker, *Dryobates villosus*	.	0.50, 0.10 (3)	.
Belted Kingfisher, *Megaceryle alcyon*	.	0.48, 0.07 (2)	.
Carolina Wren, *Thryothorus ludovicianus*	.	0.29, 0.02 (2)	.
Wood Duck, *Aix sponsa*	.	0.06, 0.49 (2)	.
Tree Swallow, *Tachycineta bicolor*	.	-0.69, 0.18 (2)	.
White-breasted Nuthatch, *Sitta carolinensis*	.	-0.31 (1)	.
Northern Cardinal, *Cardinalis cardinalis*	.	-0.34 (1)	.
Pileated Woodpecker, *Dryocopus pileatus*	.	-0.38 (1)	.
American Crow, *Corvus brachyrhynchos*	.	-0.39 (1)	.
Eastern Towhee, *Pipilo erythrophthalmus*	.	-0.65 (1)	.
Canada Goose, *Branta canadensis*	.	-0.68 (1)	.
